# Patient-Reported Outcomes Over 5 Years After Whole- or Partial-Breast Radiotherapy: Longitudinal Analysis of the IMPORT LOW (CRUK/06/003) Phase III Randomized Controlled Trial

**DOI:** 10.1200/JCO.18.00982

**Published:** 2018-12-11

**Authors:** Indrani S. Bhattacharya, Joanne S. Haviland, Anna M. Kirby, Cliona C. Kirwan, Penelope Hopwood, John R. Yarnold, Judith M. Bliss, Charlotte E. Coles, on behalf of the IMPORT Trialists

**Affiliations:** ^1^The Institute of Cancer Research, London, United Kingdom; ^2^Royal Marsden National Health Service Foundation Trust, London, United Kingdom; ^3^University of Manchester and University Hospital of South Manchester, Manchester, United Kingdom; ^4^Cambridge University, Cambridge, United Kingdom

## Abstract

**Purpose:**

IMPORT LOW demonstrated noninferiority of partial-breast and reduced-dose radiotherapy versus whole-breast radiotherapy for local relapse and similar or reduced toxicity at 5 years. Comprehensive patient-reported outcome measures collected at serial time points are now reported.

**Patients and Methods:**

IMPORT LOW recruited women with low-risk breast cancer after breast-conserving surgery. Patients were randomly assigned to 40 Gy whole-breast radiotherapy (control), 36 Gy whole-breast and 40 Gy partial-breast radiotherapy (reduced-dose), or 40 Gy partial-breast radiotherapy only (partial-breast) in 15 fractions. European Organisation for Research and Treatment of Cancer Quality of Life Questionnaires Core 30 and Breast Cancer–Specific Module, Body Image Scale, protocol-specific items, and the Hospital Anxiety and Depression Scale were administered at baseline, 6 months, and 1, 2, and 5 years. Patterns of moderate/marked adverse effects (AEs) were assessed using longitudinal regression models, and baseline predictors were investigated.

**Results:**

A total of 41 of 71 centers participated in the patient-reported outcome measures substudy; 1,265 (95%) of 1,333 patients consented, and 557 (58%) of 962 reported no moderate/marked AEs at 5 years. Breast appearance change was most prevalent and persisted over time (approximately 20% at each time point). Prevalence of breast hardness, pain, oversensitivity, edema, and skin changes reduced over time (*P* < .001 for each), whereas breast shrinkage increased (*P* < .001). Analysis by treatment group showed average number of AEs per person was lower in partial-breast (incidence rate ratio, 0.77; 95% CI, 0.71 to 0.84; *P* < .001) and reduced-dose (incidence rate ratio, 0.83; 95% CI, 0.76 to 0.90; *P* < .001) versus whole-breast group and decreased over time in all groups. Younger age, larger breast size/surgical deficit, lymph node positivity, and higher levels of anxiety/depression were baseline predictors of subsequent AE reporting.

**Conclusion:**

Most AEs reduced over time, with fewer AEs in the partial-breast and reduced-dose groups. Baseline predictors for AE reporting were identified. These findings will facilitate informed discussion and shared decision making for future patients receiving moderately hypofractionated breast radiotherapy.

## INTRODUCTION

**FIG 1.**
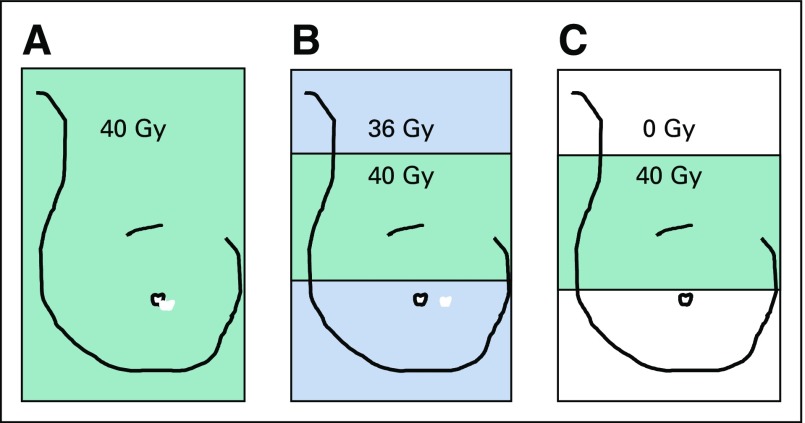
Schema of treatment groups: (A) whole-breast radiotherapy (control), (B) whole-breast and partial-breast radiotherapy (reduced-dose group), and (C) partial-breast radiotherapy only (partial-breast group).

Trials of hypofractionated whole-breast radiotherapy after breast-conserving surgery have demonstrated that 40 Gy in 15 fractions is safe and effective, with patients reporting lower levels of moderate/marked adverse effects (AEs) compared with 50 Gy in 2-Gy daily fractions.^1,2^ Consequently, the 3-week regimen tested in the START-B (UK Standardisation of Breast Radiotherapy B) trial^1^ has become standard of care in the United Kingdom for whole-breast radiotherapy and is used internationally.^3^ Subsequently, IMPORT LOW (Intensity-Modulated and Partial-Organ Radiotherapy Low Risk) investigated efficacy of partial-breast versus whole-breast irradiation using standard UK hypofractionated radiotherapy.^4^ The randomized trial schedules were: 40 Gy whole-breast radiotherapy (control); 36 Gy whole-breast and 40 Gy partial-breast radiotherapy (reduced-dose group); and 40 Gy partial-breast radiotherapy only (partial-breast group) in 15 daily fractions, using simple intensity-modulated radiotherapy (IMRT; Fig 1).

IMPORT LOW demonstrated noninferiority of partial-breast and reduced-dose radiotherapy compared with standard whole-breast radiotherapy for local relapse, with similar or fewer late normal-tissue AEs at 5 years using clinician assessments, patient-reported outcome measures (PROM), and serial photographs.^4^ These published results demonstrated that at 5 years, patients generally reported fewer moderate/marked AEs for skin changes, breast appearance change, smaller breast, and harder/firmer breast to touch in the partial-breast group compared with the whole-breast group, although the reduction was only statistically significant (*P* < .001) for change in breast appearance.^4^

This manuscript builds on the previous publication with more detailed interrogation of the large and comprehensive IMPORT LOW PROM data set. The main objective was to determine whether breast cancer treatment–related AEs improve, persist, or worsen over time to inform future patients. In addition, it was hypothesized that baseline patient-, tumor-, and treatment-specific factors could be identified that influence patterns of patient AE reporting over 5 years.

## PATIENTS AND METHODS

### Patients

The PROM substudy was conducted in a subset of IMPORT LOW, for which full details of patients and procedures have been published.^4^ All centers were invited to participate in the PROM substudy (until sufficient accrual to the substudy was achieved), and 41 of 71 centers participated. A majority of centers gave no reason for declining to participate, but a few stated lack of local research resources. There was no suggestion of a systematic difference between those centers that did and did not participate. All patients at these 41 centers were invited to participate in the substudy until the designated sample size was obtained.

### Procedures

Details of trial procedures have been published.^4^ Women were randomly assigned to receive whole-breast radiotherapy or one of the experimental schedules (reduced-dose or partial-breast).

Patients who consented to participate in the PROM substudy completed a baseline questionnaire booklet prerandomization. Subsequent questionnaires were posted by The Institute of Cancer Research Clinical Trials and Statistics Unit for completion at the patients’ homes at 6 months and 1, 2, and 5 years postrandomization. Questionnaire items investigated are summarized in Appendix Table A1 (online only).

Patients completed the European Organisation for Research and Treatment of Cancer (EORTC) general cancer scale (Quality of Life Questionnaire Core 30 [QLQ-C30]) and breast cancer–specific module (QLQ-BR23),^5,6^ Hospital Anxiety and Depression Scale (HADS; scores of 8 to 10 indicating borderline anxiety or depression, and scores of 11 to 21 indicating case levels of anxiety or depression),^7^ Body Image Scale,^8^ and protocol-specific questionnaire items.

### Statistical Methods

All analyses conducted in the whole cohort were adjusted for treatment group. The prevalence of moderate/marked (*v* none/mild) AEs at each time point was determined, and changes over time were assessed using the χ^2^ test for trend. A generalized estimating equation model^9^ including a treatment group/time interaction investigated whether prevalence of moderate/marked AEs over time differed between the treatment groups. A Poisson model adjusted for time and treatment group assessed whether average number of moderate/marked AEs per person changed over time and whether this varied according to treatment group. Separate generalized estimating equation models for each AE were fitted including terms for time and treatment group to investigate whether baseline factors predicted reporting of moderate/marked AEs over 5 years. Full details of methodology are available in the Data Supplement.

## RESULTS

**FIG 2.**
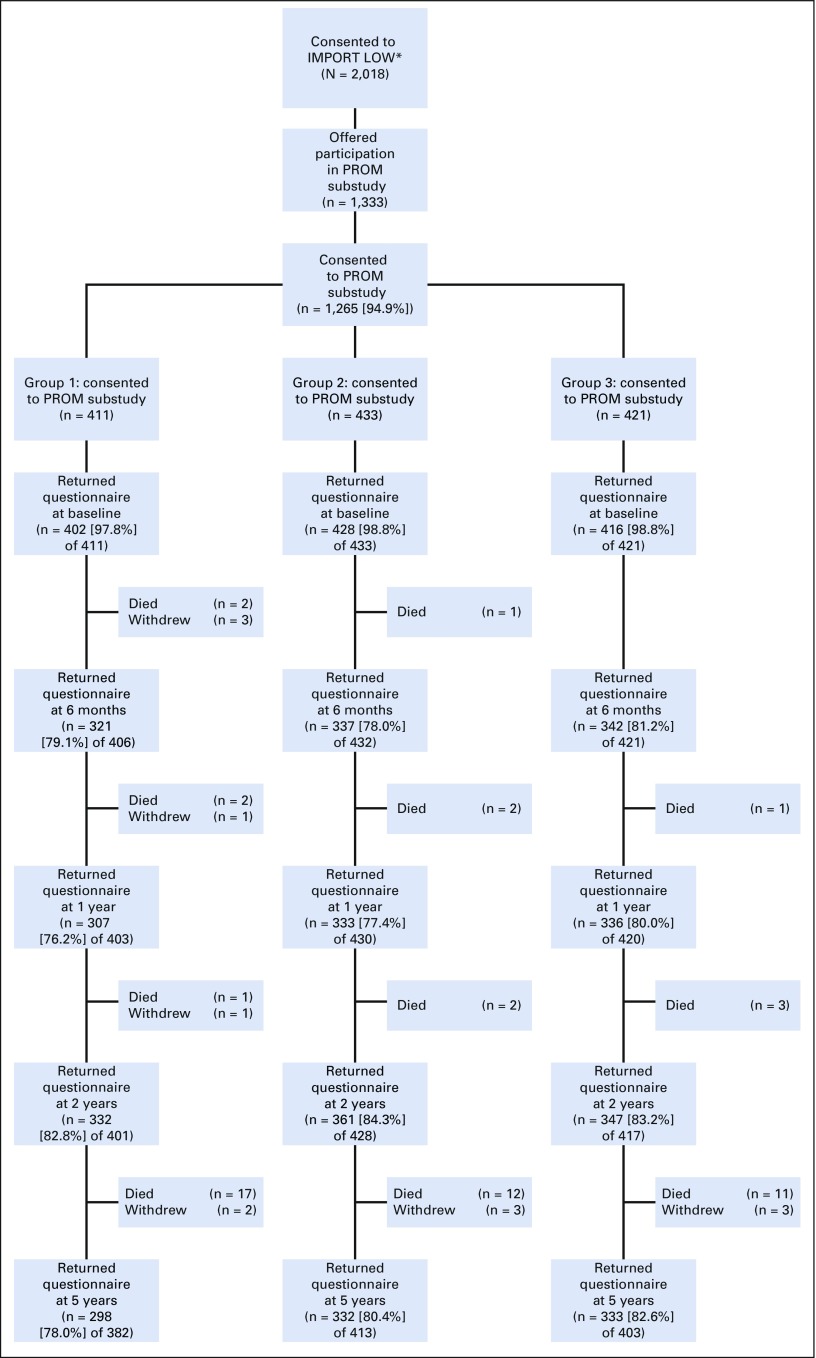
CONSORT diagram. IMPORT LOW, Intensity-Modulated and Partial-Organ Radiotherapy Low Risk; PROM, patient-reported outcome measure. (*) Two patients withdrew consent for any of their data to be used in the analysis.

A total of 2,018 women were enrolled in IMPORT LOW from 71 participating centers; 1,333 patients from 41 centers were offered participation in the PROM substudy, and 1,265 patients (95%) consented. Most women had small (approximately 1 cm), grade 1 or 2 estrogen receptor–positive and human epidermal growth factor receptor 2–negative, node-negative tumors and received adjuvant hormonal therapy (Table 1). Baseline prevalence of anxiety and depression from HADS were 23% (borderline) and 8% (case). Patients who declined the PROM study were slightly older (median age, 70 *v* 60 years); however, other baseline characteristics were similar between those who did and did not consent (Table 1). Patients who did and did not return 5-year questionnaires were similar in terms of baseline characteristics, including age, tumor characteristics, surgery details, estrogen receptor status, adjuvant therapy, and body image. There was evidence that patients who did not return their 5-year questionnaires had higher baseline HADS anxiety and depression scores than those who did (data not shown). Excluding patients who had died or withdrawn, there was a higher return rate of 5-year questionnaires in patients who reported at least one AE at 2 years (362 [85%] of 425) compared with those who reported no AEs at 2 years (601 [79%] of 764; *P* = .006). There was a high proportion of completed questionnaires at all time points (Fig 2).

**TABLE 1.**
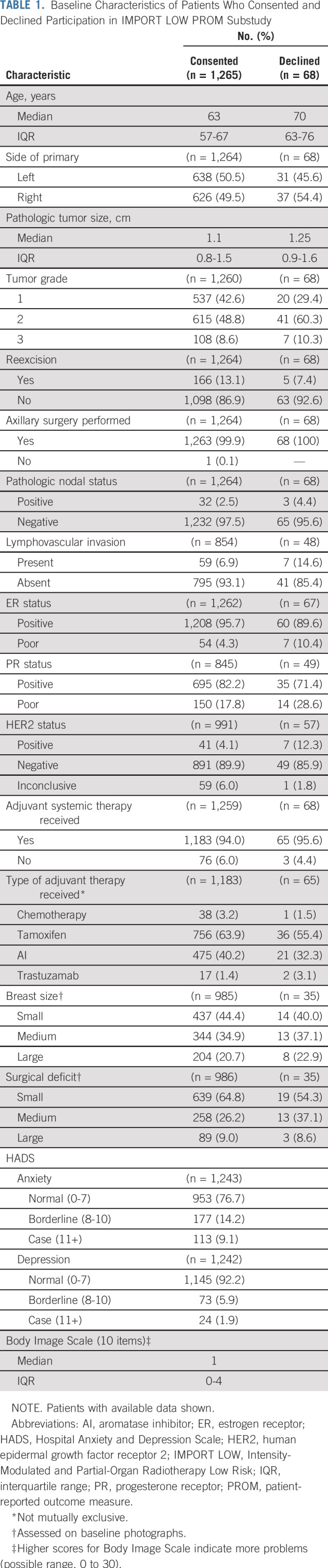
Baseline Characteristics of Patients Who Consented and Declined Participation in IMPORT LOW PROM Substudy

In the whole cohort, 557 (58%) of 962 patients reported no AEs at 5 years. Overall breast appearance change was the most prevalent AE reported at each time point and persisted over time (19% at 1 year and 21% at 5 years; Table 2). Other moderate/marked AEs, with a prevalence of greater than 10% at least once during the 5 years, were skin changes, breast hardness/firmness, breast shrinkage, nipple position affected, arm/shoulder pain, breast pain, breast swelling, and breast oversensitivity (Table 2).

**TABLE 2.**
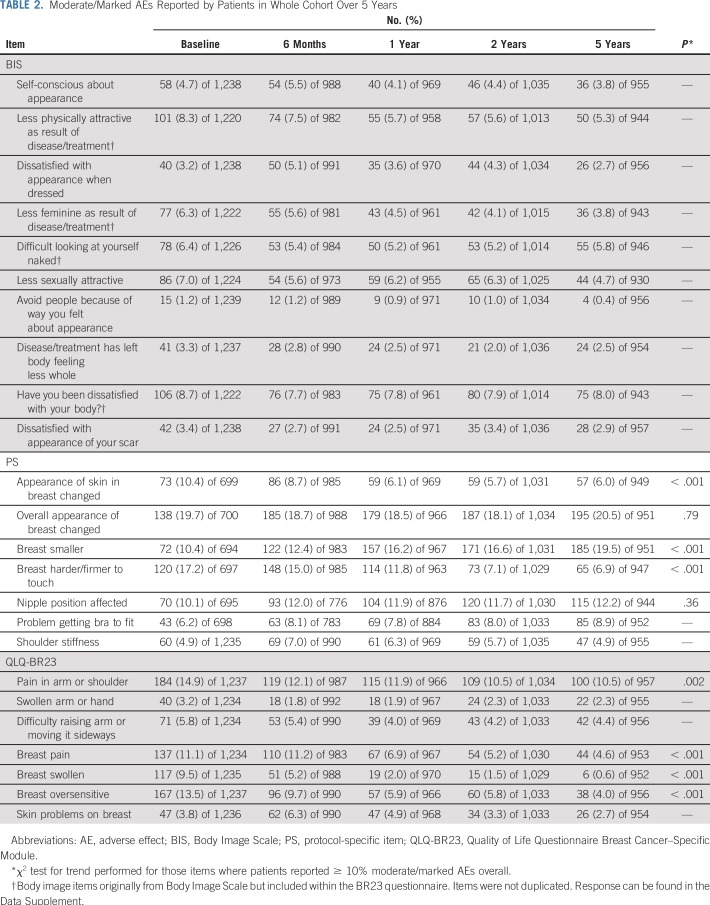
Moderate/Marked AEs Reported by Patients in Whole Cohort Over 5 Years

Overall, in patients who reported at least one AE at year 5, the median number of AEs per person at this time point was three (interquartile range [IQR], one to four); for the whole-breast group, this was three (IQR, two to five) compared with two (IQR, one to four) for both test groups. The average number of AEs reported per person at each time point was lower in the partial-breast (incidence rate ratio [IRR], 0.77; 95% CI, 0.71 to 0.84; *P* < .001) and reduced-dose (IRR, 0.83; 95% CI, 0.76 to 0.90; *P* < .001) groups compared with the whole-breast group.

**FIG 3.**
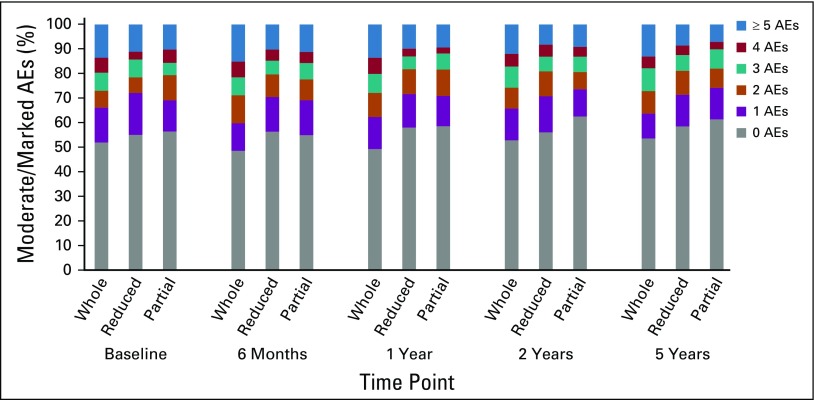
Number of moderate/marked adverse effects (AEs) reported per person over time by treatment group.

The number of AEs per person reduced over time in all treatment groups (Fig 3), at similar rates (*P* = .20). Prevalence of moderate/marked breast hardness, pain, oversensitivity, edema, skin changes (*P* < .001 for each), and arm/shoulder pain (*P* = .002) reduced over time (Table 2). Breast shrinkage was the only AE for which prevalence increased over time (*P* < .001; Table 2). There was no difference in change in prevalence of individual AEs over time between treatment groups.

Certain baseline patient factors seemed predictive for some patient-reported AEs but not others. Younger age at random assignment was associated with worse AEs for Body Image Scale items over 5 years (Table 3). In contrast, living alone was shown to be associated with reported adverse breast swelling. Education level did not predict for any AE reporting patterns (Table 4). Baseline anxiety and depression were associated with a number of AEs (Tables 3 and 4). Patients with larger breast size were more likely to report AEs (Tables 3 and 4).

**TABLE 3.**
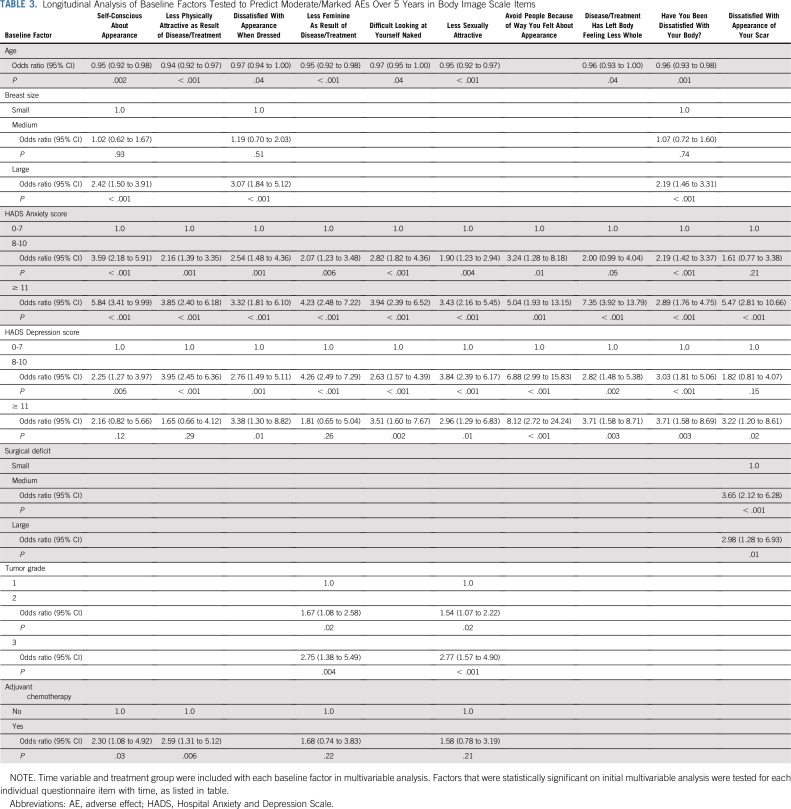
Longitudinal Analysis of Baseline Factors Tested to Predict Moderate/Marked AEs Over 5 Years in Body Image Scale Items

**TABLE 4.**
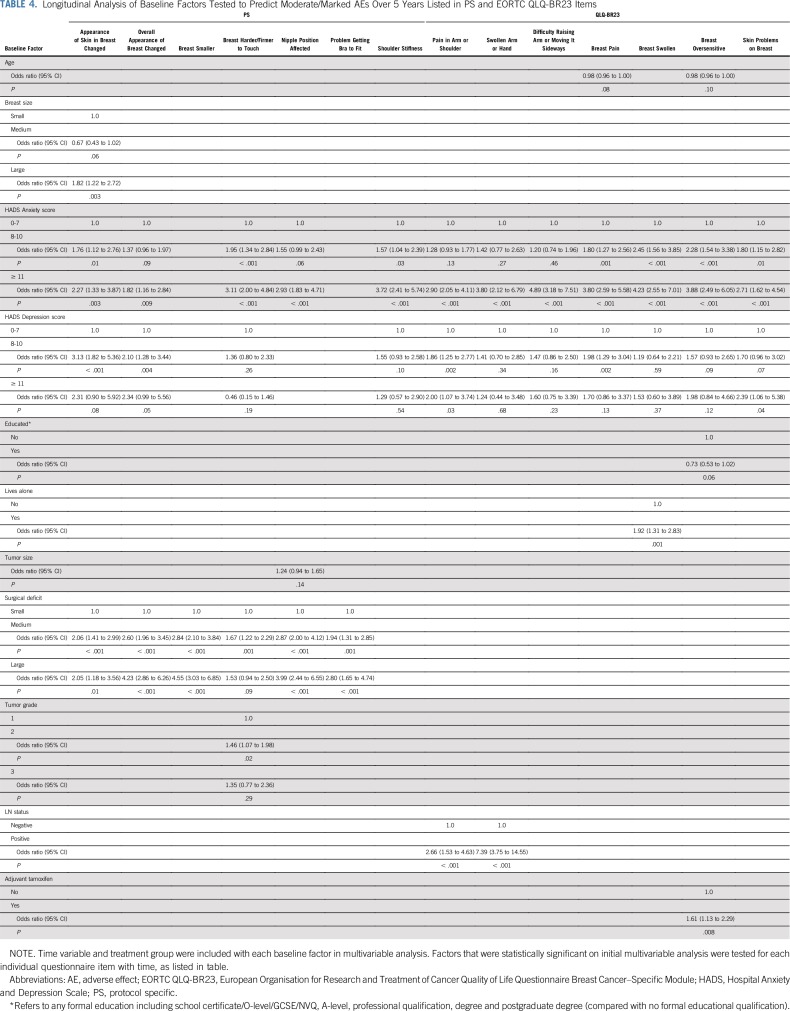
Longitudinal Analysis of Baseline Factors Tested to Predict Moderate/Marked AEs Over 5 Years Listed in PS and EORTC QLQ-BR23 Items

In relation to tumor- and treatment-specific factors, larger surgical deficit predicted for reporting of breast appearance change, breast shrinkage, nipple position affected, and problem getting a bra to fit (Tables 3 and 4). In addition, higher tumor grade was associated with reporting of feeling less sexually attractive and less feminine as a result of disease or treatment, and lymph node positivity predicted for pain in arm/shoulder and swollen arm/hand (Tables 3 and 4).

## DISCUSSION

To our knowledge, IMPORT LOW provides the largest and most comprehensive report of AEs using PROMs at serial time points from a randomized controlled trial of partial-breast radiotherapy. These data demonstrate that a majority of reported AEs reduced over time after moderately hypofractionated external-beam radiotherapy, and more than half of patients reported no moderate/marked AEs at 5 years. In addition, the average number of AEs reported per person at each time point was lower in both partial-breast and reduced-dose groups compared with the whole-breast group. Breast appearance change was the most prevalent AE reported, and this remained stable over time. All other AEs decreased over the 5-year period, with breast shrinkage, which increased, as the only exception.

Two randomized controlled trials investigating whole-breast radiotherapy at a dose of 40 Gy in 15 fractions used similar PROM assessments, which were performed at the same time points as in IMPORT LOW. These are the START-B hypofractionation trial,^10^ which compared 40 Gy in 15 fractions over 3 weeks with 50 Gy in 25 fractions over 5 weeks, and the Cambridge IMRT trial,^11^ which compared two-dimensional radiotherapy with forward planned IMRT using 40 Gy in 15 fractions in both groups. Both studies allowed a tumor bed boost at clinician discretion.

These two trials had similar PROM time trends to those shown in IMPORT LOW, because they also showed that a majority of reported AEs reduced over a 5-year period. START-B reported reduction in breast symptoms assessed using the QLQ-BR23 subscale over 5 years after radiotherapy in both standard and hypofractionation groups.^10^ The Cambridge IMRT trial^11^ reported improvement in AE reporting over the same period, but it also showed a slight initial worsening of toxicity at 6 months for skin changes, breast pain, breast oversensitivity, and breast swelling, which then improved.

A third study, the GEC-ESTRO (Groupe Européen de Curiethérapie–European Society for Radiotherapy and Oncology) trial,^12^ also incorporated PROMs using the QLQ-BR23 subscale, and assessments were performed at baseline and regularly throughout the 5-year follow-up period. Patients were randomly assigned to receive either whole-breast irradiation of 50 Gy in 25 fractions with a boost of 10 Gy or partial-breast radiotherapy using multicatheter brachytherapy. The authors found that breast symptoms assessed on the QLQ-BR23 subscale were significantly worse immediately after the last fraction of radiotherapy and at 3 months of follow-up after whole-breast versus partial-breast radiotherapy. There were no clinically significant differences between the two groups from 3 months to 5 years, and therefore, the initial worsening of symptoms in the whole-breast group were likely related to acute radiotherapy toxicity. Overall, a majority of AEs reported also seemed to decrease over time in both groups.

A fourth study, the Florence trial,^13^ used the QLQ-BR23 subscale, but only at baseline and 2 years after completion of treatment. Patients were randomly assigned to partial-breast IMRT using 30 Gy in five fractions over 1 week versus 50 Gy in 25 fractions over 5 weeks to the whole breast with an optional tumor bed boost. The partial-breast group showed improvement in PROM at 2 years, whereas breast and arm symptoms worsened in the whole-breast radiotherapy group. This difference from other reported studies may be related to the higher biologically equivalent dose with 50 Gy in 25 fractions in the whole-breast radiotherapy group compared with 40 Gy in 15 fractions in other studies. It may also reflect the smaller number of patients completing PROM questionnaires at both time points in the Florence study (205 [39%] of 520).

As per IMPORT LOW, START-B^10^ also demonstrated that breast shrinkage was the only patient-reported AE showing an increase in prevalence in the 5 years after completion of radiotherapy. The results of the Cambridge IMRT trial^11^ suggested an increase in breast shrinkage over time as reported by patients, but this did not reach statistical significance. In the START-B trial, patients reported significantly less breast shrinkage in the hypofractionation group compared with the control group.^10^ In IMPORT LOW, there was less patient-reported breast shrinkage in the reduced-dose and partial-breast groups compared with the whole-breast group; however, this did not reach statistical significance.^4^

This increase in the prevalence of breast shrinkage over time is likely to be an effect of both fibrosis and atrophy, which are recognized late normal tissue pathophysiologic consequences of radiotherapy. Therefore, the question arises of whether breast shrinkage reported by patients may be the most appropriate end point for assessing dose-volume response within breast radiotherapy trials.

IMPORT LOW showed that breast appearance change was the most commonly reported AE, and reporting remained stable over time, with significantly lower rates in the partial-breast group. Both START-B^10^ and Cambridge IMRT^11^ trials also reported breast appearance change as the most prevalent PROM, which remained stable over time. The cumulative incidence across the 5-year period in these trials was similar to that in IMPORT LOW: approximately 20% and 18% in START-B and Cambridge IMRT, respectively. This stable reporting of breast appearance over the 5-year period may reflect the dynamic interaction of some surgical changes resolving while some radiotherapy-related changes develop over time.

In contrast, different results were found in an interim analysis of the RAPID (Randomized Trial of Accelerated Partial Breast Irradiation) study,^14^ testing partial-breast radiotherapy using three-dimensional conformal radiotherapy versus whole-breast radiotherapy. Patients randomly assigned to whole-breast radiotherapy showed relatively stable reporting of breast cosmesis using the EORTC cosmetic rating system^15^ over the 5-year period, whereas those in the partial-breast group reported significantly worse cosmesis, which seemed to increase with time. The reasons for worse cosmesis in the partial-breast radiotherapy group are unclear, but it may be related to a higher biologically equivalent dose, especially if incomplete normal tissue repair after twice-daily irradiation is considered.^16^

The IMPORT LOW analysis showed that certain baseline factors were associated with some patient-reported AEs. One of these factors was younger age, within the context of a cohort of perimenopausal/postmenopausal women. This observation raised the question of whether association resulted from biologic differences (ie, differences in breast composition) or perception of AEs in the younger age group. Firstly, younger age was only associated with items in the Body Image Scale that relate to patient perception of attractiveness and sexuality. In contrast, the Cambridge IMRT trial showed that younger age was associated with increased rates of patient-reported skin changes and breast hardness^11^; however, this trial included women younger than 50 years of age, so the study population was different from that of IMPORT LOW.

Previously published results from IMPORT LOW show that there was no significant association found between age and AEs reported by clinicians or from photographs over 5 years.^4^ Similarly, in the EORTC boost versus no boost trial,^17^ age was not a predictor of clinician-assessed fibrosis. Taken together, these observations suggest that it was the perception of younger women within IMPORT LOW driving increased body image AE reporting rather than a biologic effect.

Larger breast size was a significant predictor of patient-reported AEs within IMPORT LOW. Patients with larger breasts were more likely to report skin changes and feeling self-conscious/dissatisfied with their appearance and body. In the Cambridge IMRT study, larger breast volume was also a main risk factor influencing patient-reported breast-related AEs.^11^

Larger surgical deficit predicted for increased breast appearance change and breast shrinkage in IMPORT LOW. In addition, poor baseline surgical cosmesis (related to surgical deficit) predicted for increased skin changes and breast hardness within the Cambridge IMRT trial.

Positive axillary lymph nodes predicted for worse arm/shoulder AE reporting. Similarly, the GEC-ESTRO trial reported worse arm symptom scores at baseline in patients who underwent axillary node dissection and at 3 and 6 months after whole-breast radiotherapy.^12^

In IMPORT LOW, 23% and 8% of patients were identified as being at high risk of anxiety and depression, respectively, based on baseline HADS subscale scores, and these women were more likely to report AEs. Anxiety predicted for almost all AEs, whereas association with depression was not consistently statistically significant, possibly because of a small number of patients identified as being at high risk of clinical depression. Baseline prevalence of high-risk anxiety and depression was higher in the START trials (32% and 12%, respectively) but was not investigated as a predictor of AEs.^18^ It has been reported that pretreatment psychological status may affect perception of cosmetic outcome from breast-conserving surgery and radiotherapy.^19^

Limitations include lack of baseline data regarding patient smoking, comorbidity, postoperative breast infections, and seromas, because effects of these factors were not proven to be associated with AEs during the setup of the trial. In addition, there is a possibility of reporting bias, because patients were not blinded to treatment allocation. There is an inherent risk of informative censoring with PROM questionnaire return; for example, patients with certain baseline characteristics may be more or less likely to return questionnaires. In IMPORT LOW, there were no significant differences in a majority of baseline characteristics of those who did or did not return questionnaires at 5 years, with the exception of higher baseline HADS anxiety and depression subscale scores in those who did not return their year-5 questionnaires. Bias may also arise because patients who have worse AEs may be more or less inclined to report or may represent a different subpopulation. In IMPORT LOW, we found patients who reported at least one AE at year 2 were more likely to return questionnaires at year 5; therefore, it is possible that the prevalence of AEs was overestimated in this analysis. Finally, IMPORT LOW was conducted in a lower-risk population and therefore may not be generalizable to all patients with early breast cancer.

These results demonstrate that a majority of AEs reported reduce over time. This information can provide reassurance for patients considering either whole-breast or partial-breast radiotherapy using moderately fractionated IMRT. Furthermore, baseline factors that predict AEs may be considered before radiotherapy and contribute to the informed discussion and shared clinician-patient decision-making process. Finally, this comprehensive serial analysis of PROMs adds further support to the hypothesis that partial-breast radiotherapy using moderately fractionated IMRT has less toxicity compared with whole-breast irradiation.

In conclusion, IMPORT LOW provides a unique opportunity to investigate the breadth and depth of data from a longitudinal analysis of PROMs in a large partial-breast radiotherapy trial. The results provide reassurance for future patients receiving either whole-or partial-breast moderately hypofractionated radiotherapy that treatment sequelae usually improve over time, with more than half of patients reporting no moderate/marked AEs at 5 years. Furthermore, patients receiving partial-breast radiotherapy reported fewer AEs compared with whole-breast radiotherapy using this technique. In addition, baseline factors that predict AEs can be assessed before radiotherapy, allowing tailoring of risk-benefit discussions for individuals.

## Figures and Tables

**TABLE A1.**
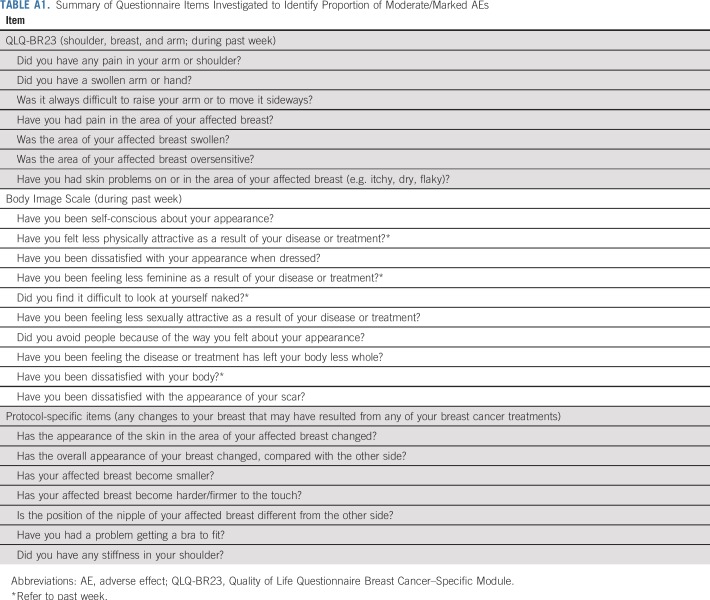
Summary of Questionnaire Items Investigated to Identify Proportion of Moderate/Marked AEs

## Appendix

The IMPORT Trialists’ Group consists of the Trial Management Group, Trial Steering Committee, Independent Data Monitoring Committee, and the principal and main coinvestigators at the participating centers.
